# Obstetrics

**Published:** 1905-06

**Authors:** W. C. Swayne


### OBSTETRICS.

**Puerperal Sepsis.**—The position occupied by midwifery, when compared with that of surgery as regards the incidence of septic infection, is one which demands serious attention. While the mortality from septic infection has been reduced to a minimum in surgical operative cases and surgical accidents, *e.g.* gunshot wounds, during the last twenty years the mortality from septic infection in midwifery is practically undiminished except in hospital practice.

The mortality from sepsis in the practice of lying-in hospitals has been reduced almost to vanishing point, while that occurring among patients delivered in their own homes shows no corresponding diminution.

Dakin[1] attributes this to the haphazard manner in which

[1] *Brit. M. J.*, 1905, i. 577.

midwifery is taught practically. He quotes tables which show that while the mortality from accidents during delivery has diminished, that from sepsis has not done so. This he believes is due to the want of sufficiently emphatic and accurate practical teaching of the technique of aseptic midwifery.

The following facts stand out in bold relief:—(1) The mortality from sepsis is practically the same as it was in 1855; (2) the mortality from sepsis in lying-in hospitals has diminished to such an enormous extent that it has almost reached vanishing point, and that not in the United Kingdom only, but in foreign clinics also; (3) the mortality in British extern maternities is almost as low as that in the intern departments.

It is hardly necessary to point out that the mortality of major surgical operations shows no such discrepancy, if operations in private are compared with those performed in hospitals. It appears obvious, therefore, that to argue, as some of those who have criticised Dakin's address have done, that numerous factors, which are common to each and all of the patients treated, have influenced the general mortality without affecting adversely either that of surgical operations, intern maternities, or extern maternities, is to disregard evidence.

Dakin compares the requirements of the licensing bodies in surgery with those in midwifery, with results which are obvious, and suggests that a maternity annexe should form part of every general hospital, in which students could receive detailed practical instruction in the same way as they are taught surgery and medicine in the wards.

Warren[1] points out that the same conditions exist in America as those mentioned as obtaining in the United Kingdom. The mortality from puerperal sepsis in general there as in this country shows no diminution, while that in hospital maternities, both extern and intern, has fallen in exactly the same way as it has in this country. In the State of Maine he shows that the mortality from sepsis was in 1902 slightly over 7%, and in the city of Portland between 3 and 4%, figures which indicate a deplorable state of things. He attributes this state of things to ignorance of the proper application of aseptic principles to midwifery, and suggests precisely the same remedy as Dakin, namely more careful education of students in the practice of midwifery, especially in aseptic technique. It is not a little striking that from opposite sides of the Atlantic the same complaint should come at the same time, and couched in terms so similar that they would almost suggest collusion if this was not obviated by the distances and dates.

The statistical preparation of Warren's paper is most thorough, but it should be read in its entirety to be properly appreciated.

Foulerton and Bonney,[2] after investigating the bacteriology of the puerperium and of puerperal infections over a period of

[1] *Am. J. Obst.*, 1905, li. 301. [2] *J. Obst. and Gynæc. Brit. Emp.*, 1905, vii. 121.

four years, report as follows (they examined altogether 96 cases):—

(1) Twelve cases in which the puerperium was normal.

(2) Fifty-four cases in which fever occurred.

(3) Thirty cases of cervicitis in non-pregnant women.

In the twelve cases in which the puerperium was normal the uterine cavity was found to be sterile.

Of the fifty-four febrile cases, in fourteen the symptoms were severe and terminated in death. In this group bacteria were found in every case, streptococcus in all but four, and B. Coli in ten.

In twenty-six cases the symptoms were severe, but all recovered; of these in five cases the uterine cavity was sterile.

Of the remaining twenty-one streptococcus occurred in nineteen.

Of the five sterile cases, however, bacterial infection of the lower vaginal tract through lacerations was proved in two of them to be streptococcal.

Of fourteen cases with slight symptoms, ten were found to be sterile as regards the uterus, but a large proportion were *primiparæ* with lacerations of the lower vagina.

Of the thirty cases of cervicitis in the non-pregnant, eleven only were sterile, but in no case was the streptococcus found.

The results show that although in many slight cases bacteria may be present, these are generally organisms of low infective power, but that in the great majority of severe cases the organisms found are those of high pathogenic intensity, and that although auto-infection is possible, yet in almost all severe cases the organism present can be identified as one introduced from without. These observations bear out similar observations of the writer in cases under his own care at various times.

The authors conclude as follows:—

That puerperal fever in the absence of proof should be treated as streptococcal.

That active curetting should be avoided.

That the best treatment for streptococcic infections is by clearing out the uterus with the finger, the use of serum and intra-uterine douches.

That bacterial invasion of the uterine cavity was marked by severe symptoms, while if the uterus was sterile but infection of the cervix or vagina had occurred, the symptoms were much less severe and the temperature did not rise above 102 deg. F.

Foulerton,[1] writing on the pathology of puerperal fevers, makes some interesting statements on the important subject of secondary infections. He states that staphylococcus aureus and albus occur but rarely as primary infections, but, the latter especially, not infrequently as secondary to streptococcal infections. Primary infections caused by S. albus., the only variety met with at all often, are always slight. B. coli communis appears to have caused primary infection in a few cases, but

[1] *Practitioner*, 1905, lxxiv., 387.

not of a severe type. As a secondary infection it is, however, very common. With regard to secondary infections with anærobic organisms, he points out that their mode of growth precludes their presence, except in cases in which either devitalised tissue is present, or in which infection with a pyogenic organism has led to venous stasis and lowered power of resistance, due to the absence of freely oxygenated blood in the tissues. When, however, infection has occurred extension is rapid owing to the virulent character of the toxins produced; these rapidly paralyse the tissues with which they come in contact, and allow of further spread of the infection if death is not caused by the violence of the toxæmia alone. He considers that infection with B. coli secondary to an infection with streptococcus does not materially increase the gravity of the disease; this is seen to be the case in similar infections of other parts, and, in addition, in cases in which serum treatment has been made use of, the symptoms have abated almost immediately in spite of the double infection.

This does not seem to apply in cases in which the secondary infection is caused by a staphylococcus.

As regards the *source* of infection, he is of opinion that the great majority of severe infections are introduced from without. This is especially the case in streptococcal infections and secondary infections with the anærobic bacilli. Auto-infection is possible in three ways: (1) through the medium of the blood from a pre-existing focus in some other part of the body, (2) from bacteria existing before labour in the cervix, or (3) in the upper or lower part of the vaginal canal. The two first, however, are the only true methods of auto-infection, since in the last the bacilli are conveyed by external agency, *e.g.* the examining finger or instruments.

As regards the uses of douches to prevent auto-infection, their efficacy seems doubtful, although they are probably harmless. The results obtained by Wormser, of Basle, indicate a very slight improvement only, as the result of preliminary disinfection of the vagina.

The treatment recommended is the use of serum and digital exploration of the uterus with intra-uterine douching. With an appropriate serum, exact diagnosis, and sufficient dosage, he has no doubt that successful treatment on these lines would become much more general.

Knyvett Gordon[1] considers, however, from his experience of severe cases of puerperal sepsis at the Monsall Fever Hospital, that infection with B. coli, even when uncomplicated, is a much more serious affection than is usually supposed. His experience, however, seems to have been obtained from cases admitted because their condition was already desperate, and not from a large number in which both slight and severe cases were included. He points out a striking fact, namely that the cavity

[1] *Ibid.*, p. 345.

of the uterus may be rendered sterile by douching, and yet the deeper layers of the decidua may teem with pyogenic organisms; he therefore looks on curetting as the proper treatment, and uses a sharp curette, with which he removes the whole of the decidua; he, however, is emphatic on the necessity of rendering sterile if possible the raw surface left. Considering that most of his cases were admitted in the second week of the disease, his percentage of recoveries must be considered good, although it is less than is usually met with in cases in which treatment has been begun early. The writer of this article showed some years ago that the percentage of recoveries falls in almost geometrical ratio as the date of the commencement of treatment is postponed after the date of attack. Gordon lost 80 per cent. of his cases.

Emery[1] refers to the value of cytological examination of the blood in cases of puerperal infection. He points out, however, that owing to the normal increase of leucocytes in pregnancy and their daily variation in the puerperium, repeated examinations are necessary. The normal leucocytosis at term is shown to be from 11,700 to 15,000 per cmm., chiefly due to increase in the polynuclears; during the puerperium the number falls daily, hence, an increase from day to day, certainly, and a cessation of decrease, probably, indicates a septic infection. A moderate and gradual rise indicates a septicæmia; a *sudden* rise is probably due to suppuration.

The fact that in the virulent or fulminating forms of infection no leucocytosis occurs is unimportant, as clinically these cases present no difficulties in diagnosis. The cases in which this examination will be found most valuable are those in which the symptoms are ill-marked, the infective process sub-acute, and the diagnosis uncertain.

Also in cases of general infection the regeneration of the red corpuscles which normally takes place in the puerperium ceases, and their number falls together with, but in a less degree than, the decrease in hæmoglobin.

The iodine reaction, or the appearance of brown granules in the staining of the protoplasm of the polynuclears, is also of value as evidence of a toxæmia.

Lockyer[2] quotes Potocki and Lacasse,[3] who have tried to settle the question as to whether blood examinations will help the clinician in puerperal fever. They consider that the prognosis is doubtful if there is great increase in the numbers of leucocytes and polynuclear cells, *e.g.* 25-30,000 of the former and 80-90 of the latter. If a marked leucocytosis is associated with a rapid diminution or disappearance of eosinophiles the prognosis is bad.

Increase in eosinophiles and diminution of leucocytosis is a sign of improvement. A rapid decrease of eosinophiles is of grave importance, and may be an indication for a radical operation.

WALTER C. SWAYNE.

[1] *Ibid.*, p. 416. [2] *J. Obst. and Gynæc. Brit. Emp.*, 1905, vii. 138.

[3] *Zentralbl. f. Gynäk.*, 1905, xxix. 31.

